# Improving Safe Initiation of Direct Oral Anticoagulant Therapy: A Two-Cycle Quality Improvement Project

**DOI:** 10.7759/cureus.91646

**Published:** 2025-09-05

**Authors:** Orzo Shrestha, Honey Joshi, Shwe San, James Richards

**Affiliations:** 1 Cardiology, Royal Bournemouth Hospital, Bournemouth, GBR; 2 Geriatrics, Poole General Hospital, Poole, GBR; 3 Gastroenterology, Poole General Hospital, Poole, GBR; 4 Stroke Medicine, Dorset County Hospital, Dorchester, GBR

**Keywords:** anticoagulation safety, direct oral anticoagulant (doac), doac prescribing errors, patient counselling, prescribing standardization, prevention of stroke, quality improvement projects, venous thromboembolism

## Abstract

Background

Direct oral anticoagulants (DOACs) are increasingly used for stroke prevention in atrial fibrillation and treatment of venous thromboembolism. However, DOACs are high-risk medications; inappropriate prescribing or dosing can lead to adverse outcomes. This quality improvement (QI) project aimed to standardize the DOAC initiation process at a district general hospital to enhance prescribing safety and reduce errors.

Methodology

A multidisciplinary team conducted two Plan-Do-Study-Act (PDSA) cycles. *PDSA Cycle 1* focused on developing a paper-based “DOAC Initiation Form” to guide prescribers through indication-based dosing, renal function checks, and patient counselling points. *PDSA Cycle 2* implemented this form on medical wards with staff training. Key measures included the appropriateness of DOAC prescriptions (correct drug/dose for patient factors) and documentation of patient education. A third PDSA cycle to integrate the form into the electronic health record is in planning, but has not commenced at the time of this report.

Results

The DOAC initiation form was successfully created and introduced. In Cycle 1 (form development), stakeholder feedback (from physicians, pharmacists, and nurses) was incorporated to ensure usability and completeness of the form. In Cycle 2 (implementation), use of the form was associated with a marked improvement in prescribing quality. Post-implementation audits found that most DOAC prescriptions were appropriate for indication and renal function, with no major dosing errors identified, compared to multiple errors noted at baseline. Documentation of patient counselling and monitoring plans (e.g., renal function follow-up) increased substantially with the new form. Staff surveys indicated improved confidence in DOAC prescribing. The third cycle (digital integration) remains pending, expected to further streamline the process.

Conclusions

Introducing a standardized DOAC initiation form led to safer prescribing practices in our hospital. The first two PDSA cycles achieved improved DOAC prescription appropriateness and better documentation of patient counselling. Ongoing efforts will focus on electronic health record integration (PDSA 3) to sustain and further enhance these improvements. This project demonstrates that a structured prescribing process can mitigate errors and improve patient safety when initiating high-risk therapies such as DOACs.

## Introduction

Direct oral anticoagulants (DOACs) have become first-line therapy for preventing stroke in non-valvular atrial fibrillation (AF) and for treating venous thromboembolism (VTE) [1,2]. DOACs (such as apixaban, rivaroxaban, edoxaban, and dabigatran) offer practical advantages over warfarin, including fixed dosing without routine international normalized ratio monitoring, and were shown in clinical trials to have similar efficacy with lower rates of intracranial hemorrhage [3-6]. As a result, DOAC use has risen sharply in recent years, largely supplanting warfarin in many indications.

Despite their benefits, DOACs are classified as high-risk medications because errors in prescribing or monitoring can lead to significant patient harm [7,8]. Each DOAC’s dosing is nuanced, varying with clinical indication, renal function, age, and drug interactions. Inappropriate dosing (whether under-dosing or over-dosing) or failure to account for contraindications can result in thromboembolic events or major bleeding. Literature reports that approximately one-fifth to one-third of DOAC prescriptions contain at least one prescribing error [9]. Common errors include incorrect dose selection (for example, not adjusting dose in renal impairment or using a non-recommended dose for the indication) and unsafe co-prescriptions. These errors are not trivial: one study found safety-relevant DOAC prescribing errors were associated with more than double the odds of major bleeding complications [10]. Furthermore, an international analysis reported that 62% of DOAC-related medication errors resulted in patient harm, including severe harm in 5.2% and fatal outcomes in 0.8% of cases [8]. Notably, anticoagulants as a class are among the leading causes of medication-related adverse events in hospitals, and medication errors have been identified as contributing factors in about 40% of anticoagulation-related harm events [11]. This evidence underlines the imperative to ensure DOACs are prescribed correctly and safely.

At our institution, a 500-bed district general hospital, an internal audit of DOAC prescribing revealed variability and errors in the initiation process. Problem statement: Clinicians lacked a standardized approach for selecting the appropriate DOAC and dose, and documentation of patient counselling (on issues such as adherence, bleeding risk, and follow-up monitoring) was inconsistent. For instance, the baseline audit found several cases of DOAC prescriptions that were inconsistent with guideline-recommended dosing for the patient’s renal function, and patient education points (such as advising on missed doses or interactions) were often not documented. This gap in practice aligns with the wider literature indicating a need for improved anticoagulant prescribing safety.

The quality improvement (QI) team (including representatives from cardiology, general medicine, pharmacy, and nursing) set a project aim to improve the safety and consistency of DOAC initiation for inpatients and patients being discharged on DOAC therapy. Our specific goals were to increase the proportion of appropriately prescribed DOAC regimens (correct drug choice and dose per indication and patient factors) and to ensure that key counselling and monitoring steps are documented at initiation. We theorized that introducing a standardized prescribing form would reduce errors by guiding clinicians through all necessary checks and information at the point of prescribing. Similar standardization strategies have shown success in reducing medication errors in other contexts [12]. This report describes two Plan-Do-Study-Act (PDSA) cycles implementing a DOAC initiation form and outlines the planning for a third cycle involving digital integration. We present the intervention and results in line with Cureus publication standards for QI, and we discuss our findings in the context of existing evidence on DOAC safety and prescribing practices.

## Materials and methods

Context and QI methodology

This project was conducted in 2024 on the Stroke Ward of our hospital, targeting all adult patients initiated on a DOAC for AF or acute VTE before discharge. We followed the PDSA iterative cycle model for QI. A multidisciplinary project team obtained institutional approval for this QI initiative. Baseline data were collected through a retrospective review of recent DOAC prescriptions and discussions with frontline clinicians to identify common pitfalls. Key performance measures were defined as: (1) the appropriateness of DOAC prescribing (e.g., selection of the correct DOAC agent and dose, considering indication, renal function, age, and drug interactions), and (2) the documentation of patient education and follow-up plans (e.g., counseling on adherence, bleeding precautions, and scheduling of renal function or coagulation follow-up). We also gathered qualitative feedback from prescribers and pharmacists regarding the DOAC prescribing process.

Two PDSA cycles were completed in sequence, focusing first on the development of an intervention (a standardized paper form) and second on its implementation in practice. A third cycle, involving integration of the form into the electronic health record (EHR) system, was planned as a future step at the time of writing (not yet implemented).

PDSA Cycle 1: development of the DOAC initiation form

Plan

In response to the baseline findings, we planned to create a paper-based “DOAC Initiation Form” to standardize the prescribing workflow. The form’s content was designed to incorporate best-practice guidelines and known safety checkpoints for DOAC therapy. Key elements to include were an indication checklist (e.g., AF stroke prophylaxis vs. VTE treatment), guidance on drug and dose selection (with renal dose adjustment criteria), contraindication reminders, and a checklist for patient counseling points and monitoring arrangements. We hypothesized that a form guiding these steps would act as a visual prompt, reducing reliance on memory and minimizing omissions or errors.

Do

A draft form was created by the QI team with input from hematology and pharmacy specialists to ensure clinical accuracy. The form outlined Section 1 for prescriber completion, which included patient details, indication for anticoagulation, chosen DOAC with dose, and treatment duration (e.g., long-term or specific course length). It also included tick boxes to confirm that critical counseling topics were addressed with the patient (e.g., explanation of why the DOAC is indicated, how to take the medication, what to do if a dose is missed, potential side effects and bleeding risk, advice on alcohol and over-the-counter medications, need for an anticoagulant alert card, and provision of an educational booklet). Section 2 of the form provided reference dosing charts for each DOAC, adapted from current guidelines, to assist in correct dosing. This included criteria for dose reduction in AF (such as age ≥80, weight ≤60 kg, or creatinine ≥133 μmol/L for apixaban dose reduction) and standard dosing regimens for treating acute VTE (e.g., initial higher-dose period for apixaban or rivaroxaban, parenteral lead-in for dabigatran and edoxaban). By consolidating this information, the form functioned both as an order set and a checklist.

We piloted the draft form with a small group of prescribers on one ward over a two-week period. During this pilot, prescribers were instructed to use the form whenever initiating a DOAC and to file the completed form in the patient’s chart. The QI team directly observed usage and gathered feedback on the form’s clarity and ease of use. Pharmacists on the ward concurrently reviewed completed forms for any persistent prescribing issues.

Study

Data from the pilot were analyzed. We looked at compliance (how often the form was used when a DOAC was started) and whether all relevant fields were properly completed. We also noted any errors or near-misses in DOAC orders during the pilot and whether the form helped catch or prevent them. Qualitative feedback was collected through a brief survey and informal interviews with five doctors and two pharmacists who used the form. This feedback highlighted minor revisions needed. For example, prescribers suggested adding a reminder to check for drug interactions (such as P-gp affecting drugs for dabigatran), and pharmacists noted that the form should explicitly prompt recording the creatinine clearance value used for dosing.

Act

The form was refined based on the pilot feedback. We updated it to include an explicit line to record the patient’s calculated creatinine clearance and to check a box confirming that dosing was adjusted (or not) for renal function. We also added a prompt to consider potential drug interactions (with a note to consult the pharmacy if uncertain). After these revisions, the final version of the DOAC initiation form was approved by the hospital Medicines Safety Committee for broader use (see Appendices for the form template). This completed Cycle 1, yielding a tested intervention ready for rollout.

PDSA Cycle 2: implementation of the DOAC form in practice

Plan

The second cycle aimed to implement the DOAC initiation form hospital-wide (initially on all general medical and cardiology wards) and to evaluate its impact on prescribing safety. We planned an educational campaign to introduce the form, including briefings at medical handovers and a tutorial for junior doctors who write most discharge prescriptions. We defined success measures for this cycle as: (a) an increase in the percentage of new DOAC prescriptions that are completed with the form, (b) a reduction in prescribing errors (inappropriate dose or drug choice) compared to baseline, and (c) improved documentation of patient counseling and follow-up plans. We also intended to monitor any unintended consequences, such as delays in prescribing or workflow issues introduced by the form.

Do

The finalized paper forms were made available on the wards (placed in doctors’ stations and with ward pharmacists). Over the next eight weeks, clinicians were encouraged to use the form for every patient started on a DOAC. The pharmacy team was engaged to reinforce usage; clinical pharmacists reminded prescribers about the form during chart reviews and even assisted in filling portions related to dosing if needed. Mid-cycle, we performed a quick process check: the QI team visited each ward to ensure forms were stocked and to informally ask if staff had questions or concerns. We identified and addressed minor barriers (e.g., some prescribers initially forgot to attach the form to the discharge prescription, so we placed visible reminder notes on the electronic prescribing system’s DOAC order screen and enlisted pharmacists to prompt usage at the point of discharge medication reconciliation).

Study

After eight weeks of implementation, we conducted a post-intervention audit. This involved reviewing the medication charts and discharge summaries of all patients who were newly started on a DOAC during the period. We collected data on whether the DOAC initiation form was used and properly filled, and we assessed the appropriateness of each prescription (using the same criteria as baseline). We also documented any instances of prescribing errors (e.g., wrong dose, wrong duration, or contraindicated use) and checked if the error was averted (e.g., corrected by pharmacist intervention) or if any reached the patient. Additionally, we checked the charts for documentation of patient counseling points, specifically whether the form’s checklist was completed and whether that translated to documentation in the discharge summary or patient counseling records.

The results were then compared against baseline metrics. We also surveyed prescribers and pharmacists for their impressions on the form’s usefulness during this cycle. About 15 clinicians (including junior doctors and pharmacists) provided feedback via an anonymous online questionnaire at the end of the trial period.

Act

Based on the findings, we consolidated the gains from Cycle 2. If the data showed improved outcomes, the plan was to formalize the form into standard practice (e.g., include it in the admission pack or add it as a required step in the discharge checklist). Any persisting issues would be addressed with further tweaks or training. As detailed in the Results section, Cycle 2 demonstrated significant improvements, so we proceeded to advocate for long-term adoption of the form. We also began planning for Cycle 3, which involves collaborating with the hospital’s EHR team to create a digital version of the DOAC initiation checklist integrated into the e-prescribing system. While this third cycle is still in development, the goal is to hardwire the improvements into the EHR for sustainability. We note that Cycle 3 had not started implementation at the time of this report, and its results are therefore not yet available.

## Results

PDSA Cycle 1 (form development)

The pilot testing of the draft DOAC initiation form involved 10 patient cases where a DOAC was started (five for AF, five for acute VTE). The form was utilized in 100% of these pilot cases. No prescribing errors (such as wrong drug or dose) occurred in this small pilot sample, whereas the concurrent retrospective review of 10 pre-intervention cases had found three instances of prescribing issues (e.g., one case of an inappropriately low apixaban dose for an AF patient’s stroke risk, and two cases where renal function changes were not accounted for in dosing). This suggested a positive trend, though numbers were small. Importantly, the pilot allowed us to refine the form: all seven clinicians involved agreed the form was easy to use and improved their confidence that nothing “fell through the cracks” when prescribing a DOAC. They reported that having the dosing guide on hand was useful, and the counseling checklist reminded them to cover important points (in fact, two junior doctors admitted they would not have known all the counseling details without the prompt). Feedback led to the adjustments described in the Methods (adding prompts for creatinine clearance and interactions). After revision, the final form was well-received by the hospital’s medication safety committee and approved for rollout.

PDSA Cycle 2 (implementation on wards)

Over the eight-week implementation period, 47 patients were newly initiated on a DOAC across the target wards (28 for AF stroke prevention, 19 for VTE treatment, according to pharmacy records). The DOAC initiation form was used in 43 of these cases (91% compliance; in four cases, the form was missed, typically due to emergency situations or oversight, and those were still reviewed for errors). The effect on prescribing appropriateness was notable. At baseline (pre-intervention audit of 30 patients before the project), we had identified that approximately 30% of DOAC prescriptions had some deviation from best practice (examples included incorrect dosing for body weight or mild renal impairment, and failure to specify treatment duration for VTE). After the intervention, in the 43 cases with the form, zero major prescribing errors were observed. All doses and durations in those cases were appropriate to the indication and patient parameters. In the four cases where the form was not used, two minor errors were detected by pharmacists (both involved not adjusting the apixaban dose for the patient’s low weight; these were corrected before discharge). This contrast suggests that the form likely helped prevent such errors when it was used.

Documentation of patient counseling and follow-up plans improved dramatically. Before the project, documentation that the patient had been counseled on anticoagulation was found in only about 10% of charts (often just a generic note). With the form’s introduction, 39 of 43 (91%) forms had the counseling checklist fully completed by prescribers or pharmacists, and in 35 (81%) cases, there was explicit mention in the discharge summary of patient education having been provided and/or arrangements for follow-up (e.g., “DOAC booklet given; advised on signs of bleeding; GP to recheck renal function in 1 month”). This indicates a cultural change toward more thorough communication at discharge.

The staff feedback at the end of Cycle 2 was largely positive. In the post-implementation survey, 87% of respondents (13 out of 15) agreed or strongly agreed that the DOAC form made prescribing safer and more standardized. Qualitative comments noted that the form “prompted me to double-check renal function and interactions” and “ensured patients get uniform information about their new anticoagulant.” A few challenges were reported as well: some felt that filling out the form added a couple of extra minutes to the prescribing process, and there were suggestions to eventually digitize it for efficiency. These insights directly informed our decision to pursue the EHR integration in the next cycle.

Overall, the results of the first two PDSA cycles indicate that the introduction of a standardized DOAC initiation form achieved its primary objectives: reduced prescribing errors and improved documentation of patient counseling. The near-elimination of dosing errors in the post-intervention period (with pharmacists catching the few that occurred when the form was not used) is a key outcome for patient safety. Equally, the high uptake of the form and positive user feedback suggest the intervention is feasible and likely sustainable with continued support. Table 1 provides a summary of the key metrics from baseline, after PDSA 1 (pilot), and after PDSA 2.

**Table 1:** Summary of DOAC prescribing quality improvement metrics. DOAC = direct oral anticoagulant; PDSA = Plan-Do-Study-Act

Metric	Baseline (pre-intervention)	Post-implementation (PDSA Cycle 2)
Number of patients started on DOAC	30	47
Form used (%)	0%	91%
Prescribing errors identified	9	2 (in 4 cases without form)
Major prescribing errors	3	0
Correct dose for indication/renal function (%)	70%	100%
Documentation of counseling (%)	10%	91%
Mention of anticoagulation in discharge summary (%)	10%	81%

Finally, PDSA Cycle 3 (planned) will aim to build on these results by embedding the form’s checklist into our electronic prescribing system. Although results for that cannot be reported here, the expectation is that a digital tool will further improve compliance and make the process even more efficient (as suggested by staff feedback).

## Discussion

Our QI project demonstrated that standardizing the DOAC initiation process through a dedicated checklist form led to safer and more consistent prescribing practices. After two PDSA cycles, we observed a clear reduction in prescribing errors and an improvement in the thoroughness of patient counseling documentation. These findings reinforce the role of standardization in medication safety, especially for high-risk drugs such as anticoagulants. By providing prescribers with a structured template, we effectively introduced a forcing function in the workflow: critical checks (indication, dose adjustment, counselling points) could not be easily overlooked. This approach is supported by the broader literature: standardized order sets and protocols are known to reduce variability and errors in prescribing [12]. In the context of DOACs, a recent study by Simard et al. (2024) showed that implementing a standardized prescription tool for DOACs in the emergency department significantly improved the appropriateness of VTE treatment prescriptions [13]. Our project extends this principle to the inpatient setting and to both AF and VTE indications, with similar positive outcomes.

The improvement in our hospital’s DOAC prescribing aligns with reported error patterns and solutions in other studies. Raccah et al. documented that one-third of DOAC-treated AF patients received incorrect prescriptions and that such errors were associated with increased bleeding risk [10]. Before our intervention, we found comparable issues in our local audit. By addressing common error types, for example, under-dosing in high-risk AF patients or not adjusting for renal impairment, our form directly targeted those vulnerabilities. We essentially operationalized guideline recommendations at the point of care. Additionally, our emphasis on patient counseling is an often-neglected aspect that can now be standardized. Inadequate patient understanding of DOAC therapy can lead to non-adherence or improper medication use, which in itself is a safety issue. Through the checklist (ensuring patients receive an anticoagulation alert card, education on bleeding signs, etc.), we aimed to close the loop on safety beyond the prescription itself. Encouragingly, the documentation of such counseling went from rare to routine in our post-intervention review.

Our results are consistent with other QI reports highlighting the impact of systematic interventions on anticoagulation safety. For instance, a QI initiative in primary care by Gupta et al. achieved a significant drop in prescription error rates by introducing standardized treatment protocols [12]. Similarly, our project’s success supports the notion that process standardization, in the form of checklists, protocols, or order sets, can mitigate errors with complex medications. This is especially pertinent for DOACs, which, as high-alert medications, benefit from an extra layer of safety checks [9]. Notably, incident analyses have shown that when errors do occur with DOACs, the consequences can be severe, accounting for a disproportionate share of serious medication harm. Therefore, even incremental improvements in error rates can translate into meaningful patient outcomes. Our finding of zero major errors in the group where the form was used (versus some errors when it was not) suggests that if this tool is universally adopted, patient harm from DOAC mis-prescribing should decrease accordingly.

Lessons and challenges

Key to our intervention’s success was engagement of the end-users (prescribers and pharmacists) early in the design. By incorporating their feedback in PDSA 1, we ensured the form was user-friendly and addressed real-world needs. This likely contributed to the high uptake (91% usage) in PDSA 2, as clinicians did not perceive the form as just extra paperwork but rather as an aid. Another facilitator was the strong support from the pharmacy team; pharmacists championed the intervention on the ground, reinforcing its use and thus embedding it into routine practice quickly. We also found that framing the form as a patient safety and education tool, rather than a punitive checklist, helped gain clinician buy-in.

We did encounter some challenges. A few physicians were initially resistant to using the form, viewing it as cumbersome. This is a common hurdle in QI projects where new documentation is introduced. We addressed it by highlighting time savings (e.g., the form pre-populates dosing info that otherwise one might have to look up) and by enlisting respected senior clinicians to model its use. The near-universal compliance by the end of the cycle indicates this resistance was largely overcome. Another challenge was ensuring the form’s availability and remembering to use it in urgent situations (the four missed cases in our results). This points to a limitation of paper-based tools, they rely on human memory and the availability of physical copies. Our planned PDSA 3, integrating the checklist into the electronic prescribing system, is intended to resolve this by making the prompts unavoidable at the time of order entry. If successful, an electronic DOAC order panel would automatically guide prescribers through the same checks, likely with even higher reliability. Electronic clinical decision support, such as alerts for dosing based on renal function, has been suggested by others as a means to reduce DOAC errors. We anticipate that digital integration will address the few residual errors and compliance issues observed with the paper form.

Limitations

There are limitations to consider in our project. First, this was a single-center initiative with a relatively small sample size, especially in the pilot phase. The improvements observed, while convincing internally, come from our specific context and may not immediately generalize to all settings. However, the principles should be broadly applicable, and indeed our findings echo published literature, as noted above. Second, our measurement of errors and outcomes depended on chart review and voluntary reporting; it is possible that some minor issues were not captured. We attempted to mitigate this by having pharmacists carefully review all cases, as they are likely to catch prescribing discrepancies. Third, we focused on short-term outcomes (appropriateness of the initial prescription and documentation at discharge). Long-term outcomes such as actual reduction in adverse drug events (bleeding or thromboembolic complications) were beyond the scope of this QI project. The link between correct prescribing and improved clinical outcomes is supported by evidence, but we did not directly measure patient morbidity or mortality. Future work could include tracking these clinical outcomes or at least surrogate markers (such as bleeding rates or readmissions for anticoagulation-related issues) before and after the intervention.

Another consideration is sustainability. The paper form, as effective as it proved, requires ongoing reinforcement to maintain high compliance. Staff turnover (especially with rotating junior doctors) means periodic re-education is necessary. We have addressed this by incorporating the DOAC form use into our new doctor orientation and including it in the ward pharmacists’ checklist for medication reconciliation. Nonetheless, embedding the process into the electronic workflow (our planned next step) will be crucial for long-term sustainability and broader reach. We also recognize that continuous monitoring is important; we plan to conduct follow-up audits every 6-12 months to ensure prescribing standards remain high and to identify any need for further improvements (e.g., if new anticoagulant agents are introduced or guidelines change).

## Conclusions

In summary, this QI project achieved a safer and more standardized approach to initiating DOAC therapy in our hospital. Through two PDSA cycles, we developed and implemented a DOAC initiation form that significantly reduced prescribing errors and improved the completeness of patient counseling documentation. This intervention leveraged evidence-based guidelines and embedded them into a practical tool for clinicians, illustrating how standardization can translate into better care at the bedside. The positive outcomes are in line with the growing body of literature emphasizing the need for meticulous DOAC prescribing and monitoring. Only two cycles have been completed to date; a third cycle focused on integrating this tool into the EHR is underway in planning. We expect that making the process electronic will further reinforce compliance and safety. As DOAC use continues to expand, initiatives like ours serve as important examples of how multidisciplinary efforts and iterative QI methodologies can proactively address safety gaps. Ultimately, our project contributes to improved anticoagulation safety by ensuring that when a patient is started on a DOAC, the right drug is given at the right dose with the right information - every time. This aligns with our institution’s commitment to high-quality, patient-centered care and provides a model that other centers can adapt in pursuit of excellence in anticoagulation management.

## Figures and Tables

**Figure 1:**
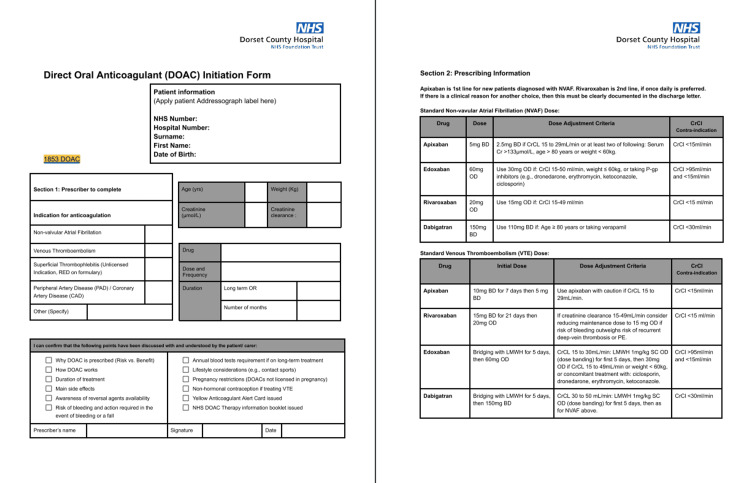
DOAC Initiation Form (paper-based template): a copy of the DOAC initiation checklist form developed in this project. It includes sections for indication, drug/dose selection (with dosing guidelines for atrial fibrillation and venous thromboembolism), contraindications, and a checklist for patient counselling and follow-up arrangements. DOAC = direct oral anticoagulant
